# Primum non nocere: shared informed decision making in low back pain – a pilot cluster randomised trial

**DOI:** 10.1186/1471-2474-15-282

**Published:** 2014-08-21

**Authors:** Shilpa Patel, Anne Ngunjiri, Siew Wan Hee, Yaling Yang, Sally Brown, Tim Friede, Frances Griffiths, Joanne Lord, Harbinder Sandhu, Jill Thistlethwaite, Colin Tysall, Martin Underwood

**Affiliations:** Division of Health Sciences, Warwick Medical School, University of Warwick, CV4 7AL Coventry, UK; Department of Primary Care Health Sciences, Radcliffe Observatory Quarter, Oxford University, OX2 6GG Oxford, UK; Universities/User Teaching and Research Action Partnership (UNTRAP), University of Warwick, CV4 7AL Coventry, UK; Department of Medical Statistics, University Medical Centre Göttingen, Humboldtallee 32, D-37073 Göttingen, Germany; Health Economics Research Group, Brunel University, UB8 3PH Uxbridge, UK; Health Professions Education Consultant, Sydney, Australia

**Keywords:** Back pain, Randomised control trial, Decision making

## Abstract

**Background:**

Low back pain is a common and disabling condition leading to large health service and societal costs. Although there are several treatment options for back pain little is known about how to improve patient choice in treatment selection. The purpose of this study was to pilot a decision support package to help people choose between low back pain treatments.

**Methods:**

This was a single-centred pilot cluster randomised controlled trial conducted in a community physiotherapy service. We included adults with non-specific low back pain referred for physiotherapy. Intervention participants were sent an information booklet prior to their first consultation. Intervention physiotherapists were trained to enhance their skills in shared informed decision making. Those in the control arm received care as usual. The primary outcome was satisfaction with the treatment received at four months using a five-point Likert Scale dichotomised into “satisfaction” (very satisfied or somewhat satisfied) and “non-satisfaction” (neither satisfied nor dissatisfied, somewhat dissatisfied or very dissatisfied).

**Results:**

We recruited 148 participants. In the control arm 67% of participants were satisfied with their treatment and in the intervention arm 53%. The adjusted relative risk of being satisfied was 1.28 (95% confidence interval 0.79 to 2.09). For most secondary outcomes the trend was towards worse outcomes in the intervention group. For one measure; the Roland Morris Disability Questionnaire, this difference was clinically important (2.27, 95% confidence interval 0.08 to 4.47). Mean healthcare costs were slightly lower (£38 saving per patient) within the intervention arm but health outcomes were also less favourable (0.02 fewer QALYs); the estimated probability that the intervention would be cost-effective at an incremental threshold of £20,000 per QALY was 16%.

**Conclusion:**

We did not find that this decision support package improved satisfaction with treatment; it may have had a substantial negative effect on clinical outcome, and is very unlikely to prove cost-effective. That a decision support package might have a clinically important detrimental effect is of concern. To our knowledge this has not been observed previously. Decision support packages should be formally tested for clinical and cost-effectiveness, and safety before implementation.

**Trial registration:**

Current Controlled Trials ISRCTN46035546 registered on 11/02/10.

**Electronic supplementary material:**

The online version of this article (doi:10.1186/1471-2474-15-282) contains supplementary material, which is available to authorized users.

## Background

A patient centred model of health care is an explicit ambition of a number of established health care providers internationally including the UK National Health Service (NHS) [1]. The NHS is making a substantial investment in promoting shared decision making across a wide range of disorders through the ‘Right Care’ programme [2]. Shared informed decision making is one important part of the patient-centred model of care. To encourage and implement informed shared decision making, decision aids for patients have been developed to support treatment choice in many areas including breast cancer [3, 4], hypertension [5] and hormonal replacement therapy [6]. These patient decision aids provide information, evidence and guidance to help patients make decisions where there is some choice and where the decision may be influenced by the patient’s values. Randomised controlled trials investigating the effectiveness of patient decision aids have shown positive effects on patient satisfaction with the decision-making process, enhanced knowledge acquisition and less decisional conflict or anxiety when making their decision on treatment preference [7–10]. In contrast, few trials have tested the effect of patient decision aids on clinical outcomes and overall these findings are not conclusive [11, 12]. The few trials that report cost outcomes provide insufficient evidence to conclude that these interventions are cost saving; none report a cost-effectiveness analysis [13].

Low back pain is a common condition, which is associated with major occupational and healthcare costs [14–17] often leading to many years lost to disability [18]. In 2009, the National Institute for Health and Care Excellence (NICE) published guidance on the management of low back pain for those suffering with the condition from six weeks to one year. These guidelines recommend that people with persistent low back pain should be offered a choice of three core treatments: manual therapy, acupuncture, or supervised group exercise [19]. The guidelines also emphasise the need for clinicians to take into account the individual’s needs and preferences as part of patient-centred care [1, 16].

Patients want more information about the available treatment options for their back pain to enable them to make better informed decisions [20–28]. Clinicians should therefore adopt a collaborative and shared approach to decision-making. This suggests a need for greater involvement by the patient in choosing the treatment they would most prefer, after a discussion with their health professional during which they have received factual information about different available options [29, 30]. The practical application and evaluation of informed shared decision making in the non-surgical treatment of low back pain has, however, been limited.

In this pilot cluster randomised controlled trial (RCT) we tested the hypothesis that the use of a decision aid together with an informed shared decision-making consultation approach would improve satisfaction with treatment for patients referred to a community physiotherapy service for treatment of low back pain. A positive signal within this pilot study would justify proceeding to a full trial to test its clinical and cost-effectiveness.

## Methods

### Trial design

The study protocol and the intervention are described in detail elsewhere [31, 32]. They are briefly summarised here. In our original study design we planned to randomise participants individually to treatment arms. Changes in the organisation of the appointment service around the time recruitment started meant we were unable to do this. We therefore, with agreement from the funder, changed to a cluster randomised design. Ethical approval was granted by Warwickshire REC (10/H1211/2).

### Participants

We recruited participants aged 18 or over who had been referred to a single community physiotherapy department in Coventry for treatment of non-specific low back pain. All participants needed to be fluent in English. We excluded participants with severe psychiatric or personality disorders, a terminal or critical illness and those with possible serious spinal pathology (e.g. tumour, sepsis or fracture).

### Interventions

Our intervention package was developed after exploratory work including literature reviews, a Delphi study, a nominal group with physiotherapists, focus groups with patients’ and secondary analysis of existing interview data. We have described this process in detail elsewhere [32]. We developed a patient decision support package in the form of a booklet and associated training for the physiotherapists [32] (Additional file 1). The physiotherapist training was a two-hour session to enable the therapists to develop an understanding of how to incorporate the decision support package within their patient-centred consultations, facilitate patient involvement as well as providing appropriate information by recognising and responding to patient concerns.

The intervention consisted of a patient booklet that details the available treatment options which include exercise, manual therapy, acupuncture, and a cognitive behavioural approach. The booklet also provided answers to the frequently asked questions associated with each option. Space was provided in the booklet to enable patients to note if they felt they had sufficient information and any points they wanted to discuss in the consultation. This was posted ahead of the participant’s first consultation and used as a basis for discussion when they attended.

Those allocated to a therapist in the usual care arm attended their appointment without any prior treatment information and with a therapist not trained on informed shared decision making.

### Procedure

Once a general practitioner referral was received, patients were given an appointment by a booking clerk who was blind to the physiotherapists’ allocation. These patients were then sent an invitation to join the study. Each invitation pack contained an invitation letter, patient information sheet, consent form, baseline questionnaire and reply slip. The invitation advised participants that the trial was looking at whether giving physiotherapists additional training will improve their satisfaction with their choice of back pain treatments. Participants were advised that they would be randomly assigned to either a specially trained physiotherapist or a physiotherapist who had not had the additional training. Potential participants returned the consent form and completed baseline questionnaire to the research team at Warwick Clinical Trials Unit. Those who met the inclusion criteria, completed the baseline questionnaire and consented to the trial were included.

Once the baseline questionnaire was received those participants allocated to a therapist in the intervention arm of the study were sent a copy of the decision support booklet by post from the physiotherapy department; typically around two weeks prior to their first consultation [32]. Participants were then expected to attend their appointment as usual. Those allocated to a therapist in the usual care arm attended their appointment as usual.

### Outcomes

The primary outcome measure was satisfaction with treatment at four months using a five-point Likert Scale (very satisfied to very dissatisfied) dichotomised into “satisfaction” (very satisfied or somewhat satisfied) and “non-satisfaction” (neither satisfied nor dissatisfied, somewhat dissatisfied or very dissatisfied).

Participants were sent a satisfaction with decision scale [33] to complete soon after the planned initial consultation. Other secondary outcome measures collected at four months were the Roland Morris Disability Questionnaire [34], modified Von Korff pain and disability scales [35], SF-12 [36], EQ-5D [37], hospital anxiety and depression scale [38], pain self-efficacy questionnaire [39], and the fear avoidance beliefs questionnaire [40].

Outcome measures were collected at baseline and four months after entering the trial by which time we would expect all treatment to have been completed and any positive effects maximised. In addition we sent an immediate follow-up questionnaire looking at satisfaction with decision soon after the date of their first appointment. We sent two postal reminders; if necessary this was followed by a phone call from the study team member, who was blind to treatment allocation, to collect the primary outcome and modified Von Korff scales [35].

### Sample size

To show an improvement of 25% in satisfaction with treatment from 50% in the control arm at the 5% significance level with 80% power requires data on 116 subjects. Although such satisfaction measures are prone to ceiling effect we have used a dichotomised scale. Allowing for 20% loss to follow-up we originally aimed to recruit 150 subjects. With the change in design (from individual randomised to cluster randomised trial) we needed to account for clustering effects. We did a blinded interim sample size review [41] after we had recruited 41 participants to estimate the intra-cluster correlation coefficient (ICC) to allow a re-calculation of sample size. The estimated ICC was close to zero and there were 14 clusters with size ranges from one to six. Thus, the revised sample size was based on 14 clusters with an average cluster size of nine and assuming, conservatively, an ICC of 0.01, a 5% significance level and 80% power, and allowing a 20% of loss to follow-up, the total sample size needed was 158.

### Randomisation

Using stratified block randomisation all physiotherapists working in the service were randomised to deliver either the usual care or the decision support package intervention in a 1:1 ratio, stratified by their number of years of experience (≤6 vs. > 6 years since their qualification) and the amount of time they worked for the service each week. The randomisation was set up by an independent statistician. Members of the team were blind to the allocation of participants to physiotherapists. Practical constraints meant that we were unable to train and randomise any new physiotherapists joining the service after the trial had started. Participants consulting the newly started physiotherapists were all assigned to control.

### Statistical methods

The statistical team (SWH, TF) developed a statistical analysis plan that was agreed by the study team prior to a database freeze and unblinding of the data. The main summary and analysis were intention to treat. Continuous variables at baseline and follow-ups were summarised as mean, standard deviation, median and interquartile range. Categorical variables were summarised as frequency and percentage. The primary outcome, satisfaction with treatment, was dichotomised into “satisfaction” and “non-satisfaction.” Secondary outcomes were summarised as change from baseline. The ICC and its confidence interval for the primary outcome was also summarised [42]. Dichotomous outcomes were modelled with generalised linear mixed effects regression with logit link and continuous outcomes were analysed using Gaussian linear mixed effects models. For ease of interpretation results were transformed from the odds ratio to the relative risk scale. The estimation was obtained in the same manner as for odds ratio but with log link. The study arm, physiotherapists’ years of experience (≤6 vs. >6 years) and the pain severity (modified Von Korff pain score measured at baseline) formed the fixed effects whilst the physiotherapists (clusters) formed the random effects. In a sensitivity analysis physiotherapists recruited post-randomisation were excluded. All statistical analyses were two-sided and point estimates were reported with the corresponding 95% confidence interval.

Cost-effectiveness analysis was conducted in line with the NICE reference case [43] and International Society for Pharmacoeconomics and Outcome Research recommendations [44]. We estimated the total cost of back pain related NHS care over four months. Costs for the decision support package included printing and delivery of the package to patients, and training for physiotherapists. Participants’ use of other NHS services for back pain was obtained from the follow-up questionnaire and physiotherapy records. Unit costs for health services were identified from standard national sources. The effectiveness of the decision support package and usual care were measured using quality adjusted life years (QALYs), estimated using the ‘area under the curve’ approach from EQ-5D data.

Missing resource use and utility data were imputed and integrated in the cost-effectiveness analysis using multiple imputation methods. The Stata Multivariate Imputation using Chained Equations (MICE) procedure was used to estimate missing resource use and EQ-5D observations based on patients’ age, gender, baseline RMDQ score and treatment group. Between-group differences in mean costs and QALYs were estimated using bootstrap regression of the imputed datasets. The seemingly unrelated regression method [45] was used to simultaneously estimate differences of costs and QALYs between treatment arms, adjusting for patient age, sex, Roland Morris Disability Questionnaire and EQ-5D scores at baseline, and physiotherapist experience. A simple model was used to extrapolate between-group differences in QALYs and costs (excluding the cost of the decision support package itself) at the end of the trial to a maximum of one year from baseline. This was based on the results of a published meta-analysis of low back pain trial data [46]. Analyses were done in SAS 9.3 and Stata 11.0.

## Results

A total of 19 physiotherapists were involved in the trial. Twelve physiotherapists were present at the start of the trial, and seven were randomised to the decision support arm. Of the seven physiotherapists in the intervention arm, four had more than six years of experience, whereas of the five who were randomised to the control arm only one of them had more than six years of experience (Table 1). The other seven physiotherapists who joined the department after randomisation were allocated to the control arm and four of them had more than six years of experience.

**Table 1:** **Physiotherapists and patients by treatment arms and physiotherapists’ years of experience**

		Treatment arms		
Years of experience		Intervention	Control	Control (post-randomization)
<= 6 years	No. of physiotherapists	3	4	3
	No. of patients	38	23	10
	Median	12	6	1
	(min, max)	(12, 14)	(3, 8)	(1, 8)
> 6 years	No. of physiotherapists	4	1	4
	No. of patients	30	10	8
	Median	8.5	10	1
	(min, max)	(2, 11)	(10, 10)	(1, 5)

**Figure 1:**
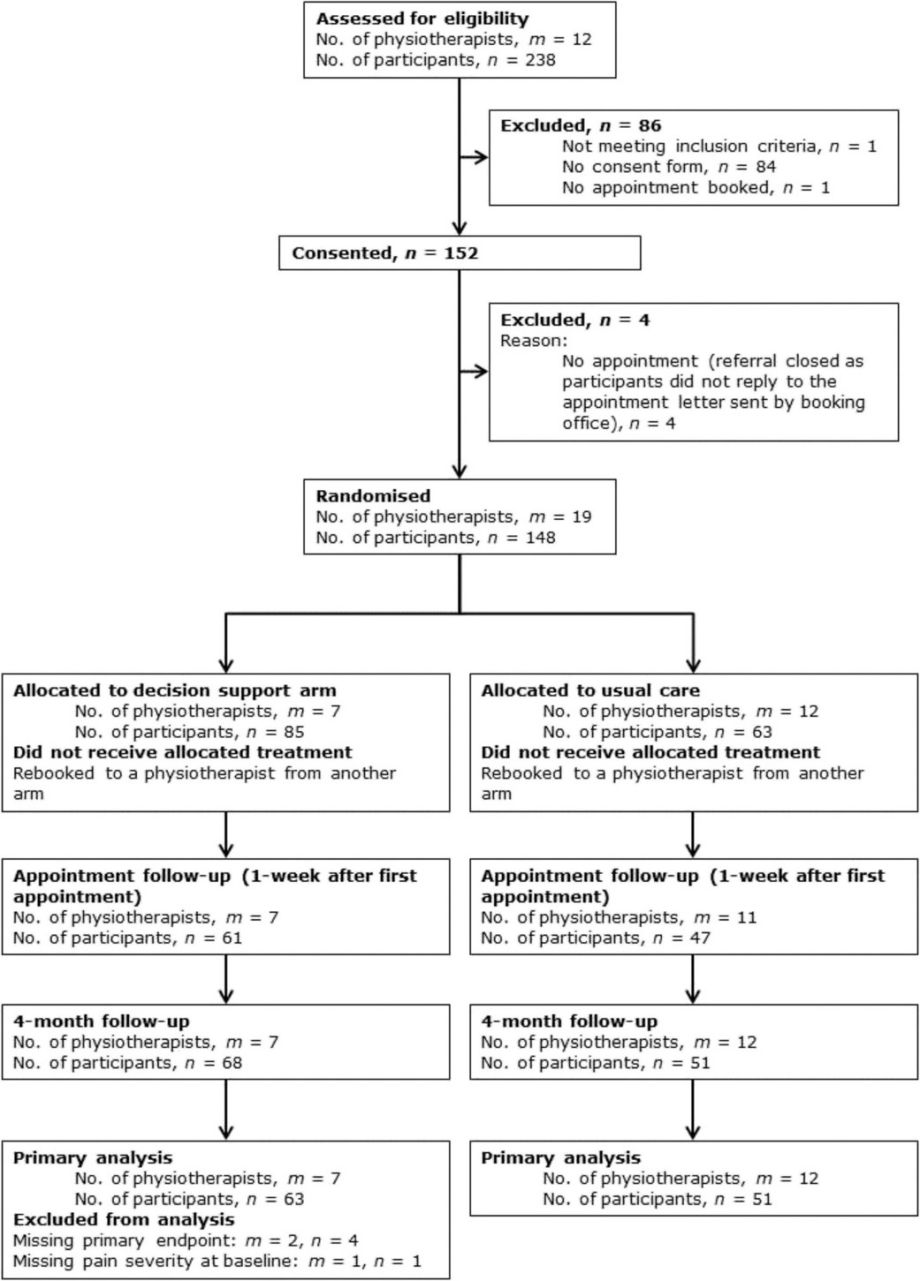
**CONSORT chart.**

We approached 238 people to take part in the trial; 148 (62%) joined the study. The baseline characteristics of the two groups were similar (Table 2). Eighty-five (57%) participants were assigned to the decision support arm. We obtained four–month follow-up data on 119 (80%) of our participants (Figure 1). Of these 114 had complete data for both primary endpoint and baseline data for analysis. The adjusted percentage satisfied with their treatment in the intervention arm was 53% (n = 33/63) and in the control arm it was 67% (n = 34/51). The adjusted odds ratio was 1.85 (95% confidence interval 0.65 to 5.25: the estimated ICC was 0.018; -0.11 to 0.15). Correspondingly, the adjusted relative risk was 1.28 (0.79 to 2.09). We infer that the rate of satisfied with treatments in the control arm was between 0.79 and 2.09 times that for those in the intervention arm.

**Table 2:** **Demographic and clinical characteristics of respondents at baseline**

	Decision support package		Usual care	
	*n*	(%)	*n*	(%)
No. of participants	85	(57.4)	63	(42.6)
Age, years				
Mean (SD)	46.9	(13.8)	48.8	(16.7)
Median (IQR)	47.6	(36.1, 56.9)	46.8	(33.5, 63.4)
Sex:				
Male	28	(32.9)	22	(34.9)
Female	57	(67.1)	41	(65.1)
Ethnicity:				
White	71	(83.5)	56	(88.9)
Mixed	3	(3.5)	2	(3.2)
Asian or Asian British	7	(8.2)	3	(4.8)
Black or Black British	3	(3.5)	2	(3.2)
Chinese	1	(1.2)	0	(0.0)
Currently working:				
Yes	45	(52.9)	35	(55.6)
No:	40	(47.1)	28	(44.4)
Retired	13	(31.7)	14	(46.7)
Stay at home	4	(9.8)	3	(10.0)
Unable to work due to low back pain	8	(19.5)	8	(26.7)
Unable to work due to other illness	6	(14.6)	2	(6.7)
Unemployed and looking for work	3	(7.3)	1	(3.3)
In full time education	2	(4.9)	0	(0.0)
Others	5	(12.2)	2	(6.7)
Hours of paid work per week:				
No. of participants	49		38	
Mean (SD)	31.8	(12.6)	30.2	(12.4)
Median (IQR)	37	(25, 40)	35	(21, 39)
Pain in lower back pain in the last four weeks:				
Yes	85	(100.0)	63	(100.0)
If Yes:				
Pain limit usual activities for more than one day:				
Yes	70	(84.3)	54	(85.7)
No	13	(15.7)	9	(14.3)
Frequency of lower back pain:				
On some days	4	(4.7)	4	(6.4)
On most days	13	(15.3)	12	(19.1)
Everyday	68	(80.0)	47	(74.6)
The length of time since free of low back pain for one whole month:				
< 3 months ago	23	(27.4)	21	(33.3)
≥ 3 months ago but < 7 months ago	18	(21.4)	5	(7.9)
≥ 7 months ago but < 3 years ago	26	(31.0)	24	(38.1)
≥ 3 years ago	17	(20.2)	13	(20.6)
Pain down the leg:				
Yes	62	(72.9)	46	(73.0)
No	23	(27.1)	17	(27.0)
If Yes:				
Pain spread below the knee:				
Yes	38	(62.3)	27	(58.7)
No	23	(37.7)	19	(41.3)
Roland Morris disability questionnaire (0–24, 0 = best)				
No. of participants	85		63	
Mean (SD)	10.66	5.9	10.6	5.3
Median (IQR)	11.0	(6.0, 14.0)	11.0	(6.0, 14.0)
Modified Von Korff disability score (0–100, 0 = best)				
No. of participants	84		62	
Mean (SD)	61.3	23.9	56.8	26.0
Median (IQR)	65.0	(48.3, 80.0)	58.3	(40.0, 76.7)
Modified Von Korff pain score (0–100, 0 = best)				
No. of participants	84		63	
Mean (SD)	68.5	20.3	67.1	17.2
Median (IQR)	73.3	(53.3, 83.3)	70.0	(56.7, 80.0)
Norm-based physical component score of SF-12 (0–100, 100 = best)				
No. of participants	85		55	
Mean (SD)	34.6	9.5	35.1	9.0
Median (IQR)	33.5	(27.2, 42.8)	34.3	(29.0, 40.0)
Norm-based mental component score of SF-12 (0–100, 100 = best)				
No. of participants	85		55	
Mean (SD)	42.6	12.8	44.4	12.2
Median (IQR)	42.7	(34.8, 53.6)	44.9	(37.2, 54.2)
Hospital anxiety and depression scale: anxiety (0–21, 0 = best)				
No. of participants	83		60	
Mean (SD)	7.6	4.4	7.5	4.2
Median (IQR)	7.0	(4.0, 11.0)	7	(4.5, 11.5)
Hospital anxiety and depression scale: depression (0–21, 0 = best)				
No. of participants	82		59	
Mean (SD)	7.2	4.7	7.2	5.0
Median (IQR)	6.5	(4.0, 10.0)	7	(3.0, 11.0)
Pain self-efficacy questionnaire (0–60, 60 = best)				
No. of participants	82		61	
Mean (SD)	34.2	15.6	31.5	14.8
Median (IQR)	37.0	(24.0, 44.0)	32.0	(19.0, 45.0)
Fear avoidance beliefs questionnaire (0–24, 0 = best)				
No. of participants	85		59	
Mean (SD)	14.3	5.9	15.5	5.5
Median (IQR)	15	(10.0, 18.0)	17	(12.0, 19.0)

*Abbreviations*: *SD* standard deviation; *IQR* interquartile range.

**Figure 2:**
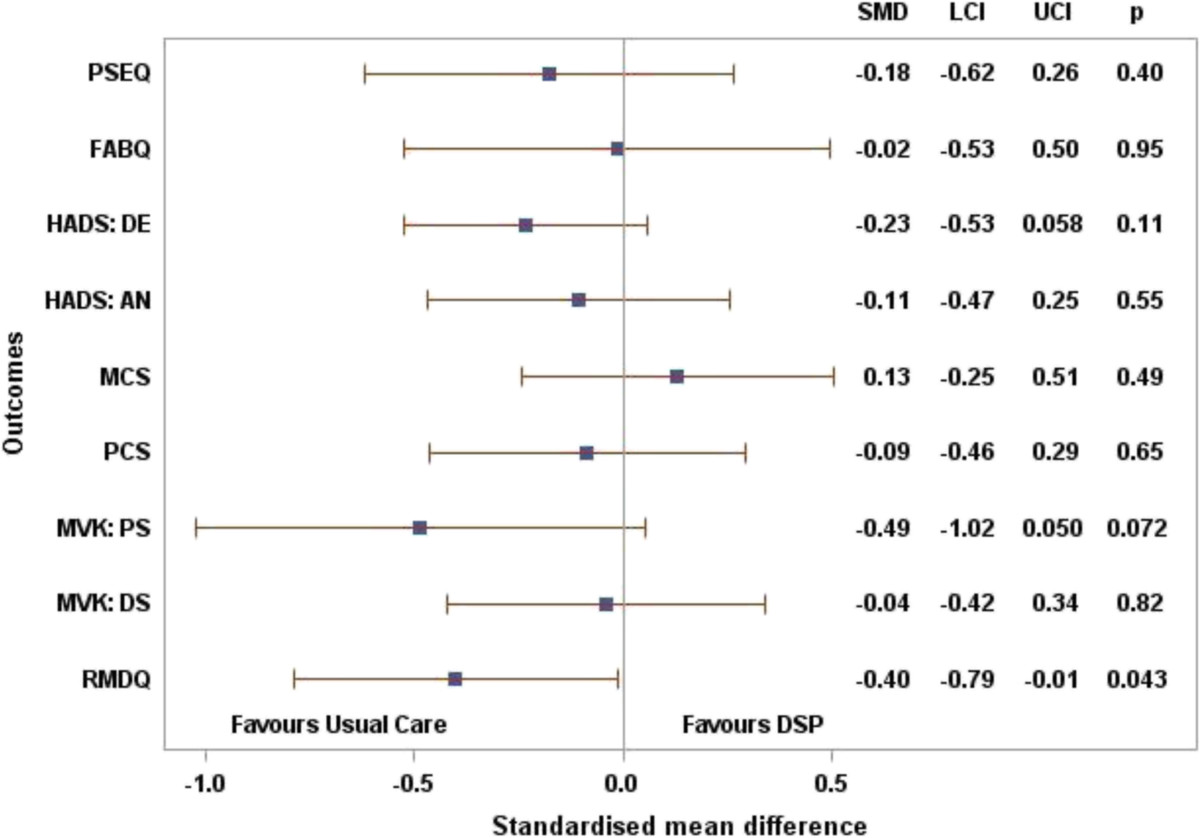
**Secondary outcomes; standardised mean difference with 95% confidence interval after adjusting for physiotherapist’s years of experience and pain severity at baseline as fixed effects and physiotherapist as random effects.**

For all but one of our secondary outcomes the point estimate for the difference favours the control intervention. The exception is the SF-12 mental component scores (Table 3, Figure 2). For the Roland Morris Disability Questionnaire the difference between treatment arms was -2.27 (-4.47 to -0.08).

**Table 3:** **Secondary outcome measures; number of participants contributed to the analysis (first row), mean change from baseline and difference between treatments with 95% confidence interval**

Mean change from baseline		Mean treatment difference*
Decision support package	Usual care	
Roland Morris disability questionnaire (positive change = improvement)		
*n* = 58	*n* = 40	
1.9 (0.5 to 3.3)	4.2 (2.5 to 5.9)	-2.27 (-4.47 to -0.08)
Modified Von Korff disability score (positive change = improvement)		
*n* = 64	*n* = 49	
17.1 (10.9 to 23.3)	18.2 (11.0 to 25.3)	-1.07 (-10.50 to 8.37)
Modified Von Korff pain score (positive change = improvement)		
*n* = 66	*n* = 50	
14.7 (7.6 to 21.9)	24.0 (16.6 to 31.4)	-9.24 (-19.44 to 0.96)
Norm-based physical component score of SF-12 (negative change = improvement)		
*n* = 53	*n* = 35	
-5.5 (-7.7 to -3.3)	-6.3 (-9.1 to -3.6)	0.80 (-2.71 to 4.32)
Norm-based mental component score of SF-12 (negative change = improvement)		
*n* = 53	*n* = 35	
-3.4 (-6.4 to -0.4)	-1.8 (-5.5 to 1.9)	-1.64 (-6.36 to 3.09)
Hospital anxiety and depression scale: anxiety (positive change = improvement)		
*n* = 57	*n* = 36	
0.5 (-0.5 to 1.5)	1.0 (-0.2 to 2.2)	-0.46 (-2.01 to 1.09)
Hospital anxiety and depression scale: depression (positive change = improvement)		
*n* = 55	*n* = 36	
1.1 (0.2 to 2.0)	2.2 (1.1 to 3.3)	-1.12 (-2.52 to 0.28)
Pain self-efficacy questionnaire (negative change = improvement)		
*n* = 57	*n* = 38	
-4.6 (-9.4 to 0.2)	-7.3 (-12.2 to -2.5)	2.74 (-4.04 to 9.51)
Fear avoidance beliefs questionnaire (positive change = improvement)		
*n* = 56	*n* = 36	
2.0 (0.2 to 3.9)	2.1 (-0.2 to 4.4)	-0.09 (-3.04 to 2.87)

*Mean difference = (decision support package – usual care) after adjusting for years of experience and pain severity at baseline as fixed effects and physiotherapist as random effects; negative difference = favours usual care.

The responses to the satisfaction with decision questionnaire were dichotomised to “agreement” and “non-agreement.” A total of 103 participants returned the immediate follow-up questionnaire. Participants from both arms were similarly satisfied with their decision (Table 4).

**Table 4:** **Summary of satisfaction with decision**

	Decision support package		Usual care	
	*n*/*N*	(%)	*n*/*N*	(%)
Satisfied that I was adequately informed about the issues important to treatment decision				
Agreement*	45/58	(78)	37/45	(82)
The decision I made was the best decision possible for me personally				
Agreement*	46/58	(79)	35/45	(78)
Satisfied that my decision was consistent with my personal values				
Agreement*	47/57	(83)	36/44	(82)
Expect to successfully carry out (or continue to carry out) the decision I made				
Agreement*	48/58	(83)	37/43	(86)
Satisfied that this was my decision to make				
Agreement*	46/58	(79)	34/43	(79)
Satisfied with my decision				
Agreement*	47/58	(81)	35/43	(81)

*Agree, and strongly agree.

The estimated cost of the decision support intervention was £19.44 per person (comprising £2.20 for consumables and £17.24 for physiotherapist training). Participants’ use of NHS services for back pain and unit costs for those health services were presented in Table 5 and Additional file 2: Table S1. Mean estimated costs for other NHS services related to back pain over the trial period in the intervention (n = 45) and control (n = 35) arms were £245 and £271, respectively (mean difference £25.76, -116.54 to 168.05) (Additional file 3: Table S2). Mean QALYs gained on the basis of EQ-5D data (Table 6) over the four-month trial period were lower for the decision support arm (n = 57) than usual care (n = 38) arm: 0.18 and 0.22, respectively (mean difference 0.03, -0.01 to 0.07).

**Table 5:** **Mean NHS service use: 0–4 months**

	Number of patients		Mean quantity		Mean treatment difference (95% CI) (usual care - DSP)
	DSP	Usual care	DSP (SD)	Usual care (SD)	
**NHS Services**					
General Practitioner	57	39	1.4(2.1)	1.1(1.5)	-0.28(-1.05 to 0 .50)
Practice nurse	58	39	0.1(0.4)	0.03(0.2)	-0.08(-0.21 to 0.06)
Physiotherapist visit**	81	60	3.8(3.7)	3.1(3.7)	0.72(-1.97 to 0.52)
Doctor/nurse in an emergency department(casualty)	58	39	0.2(0.7)	0.1(0.3)	-0.10 (-0.34 to 0.13)
Hospital specialist (consultant or team member)	58	39	0.1(0.5)	0.5(1.5)	0.42 (-0.00 to 0.84)
Psychologist/counsellor	58	39	0.03 (2.3)	0.1(0.5)	0.04 (-0.11 to 0.19)
Hospital stay	57	40	0.04(0.2)	0(0)	-0.04(-0.09 to 0.02)
**NHS Tests**					
X-rays	58	39	0.1(0.3)	0.0(0.2)	0.03(-0.14 to 0.07)
CT scan	58	39	0.02(0.1)	0.03(0.2)	0.01(-0.05 to 0.07)
MRI scan	57	39	0.1(0.3)	0.3(0.6)	0.16 (-0.04 to 0.36)
Blood tests	58	39	0.2(0.5)	0.1(0.3)	0.05 (-0.22 to 0.11)
**NHS Drugs**					
Pain killers	57	39	1.2(2.0)	1.3(1.5)	0.08 (-0.67 to 0.83)
Anti-inflammatory drugs	58	39	0.8(1.3)	0.9(1.2)	0.08 (-0.43 to 0.58)
Gels/creams	58	39	0.2 (0.5)	0.1(0.2)	0.11(-0.28 to 0.07)
Sleeping pills	58	39	0.1(0.5)	0.1(0.4)	0.04 (-0.24 to 0.16)
Anti-depressants	58	39	0.2(0.7)	0.4(0.9)	0.17 (-0.16 to 0.50)

**Based on physiotherapist’s medical record.

**Table 6:** **Utility weights and quality-adjusted life years estimates**

	Number of patients		Mean (SD)		Mean treatment difference (95% CI) (Usual care – DSP)
	DSP	Usual care	DSP	Usual care	
EQ-5D:					
Baseline	84	61	0.4 (0.4)	0.5 (0.3)	0.01 (-0.10 to 0.12)
4 month	58	39	0.5 (0.4)	0.7 (0.3)	0.13 (-0.01 to 0.26)
Change from baseline to 4-month	57	38	0.1 (0.3)	0.2 (0.3)	0.05 (-0.05 to 0.16)
QALY gains (AUC 0–4 month)	57	38	0.2 (0.1)	0.2 (0.1)	0.03 (-0.01 to 0 .07)
SF-6D:					
Baseline	85	59	0.6 (0.1)	0.6 (0.2)	0.02 (-0.03 to 0.06)
4 months	57	39	0.7 (0.2)	0.7 (0.2)	0.03 (-0.03 to 0.10)
Change from baseline to 4-month	57	37	0.1 (0.1)	0.1 (0.2)	0.03 (-0.03 to 0.08)
QALY gains (AUC baseline to 4-month)	57	37	0.2 (0.1)	0.2 (0.1)	0.01 (-0.01 to 0 .03)
EQ-VAS:					
Baseline	83	60	54.7 (26.3)	59.0 (20.6)	4.35 (-3.72 to 12.42)
4 months	55	37	68.0 (22.6)	67.7 (18.3)	-0.28 (-9.14 to 8.59)
Change from baseline to 4-month	54	36	9.0 (21.0)	8.1 (15.9)	-0.98 (-9.16 to 7.20)

*Abbreviations*: *DSP* decision support package; *SD* standard deviation; *CI* confidence interval; *QALY* quality-adjusted life years; *AUC* area under the curve.

**Figure 3:**
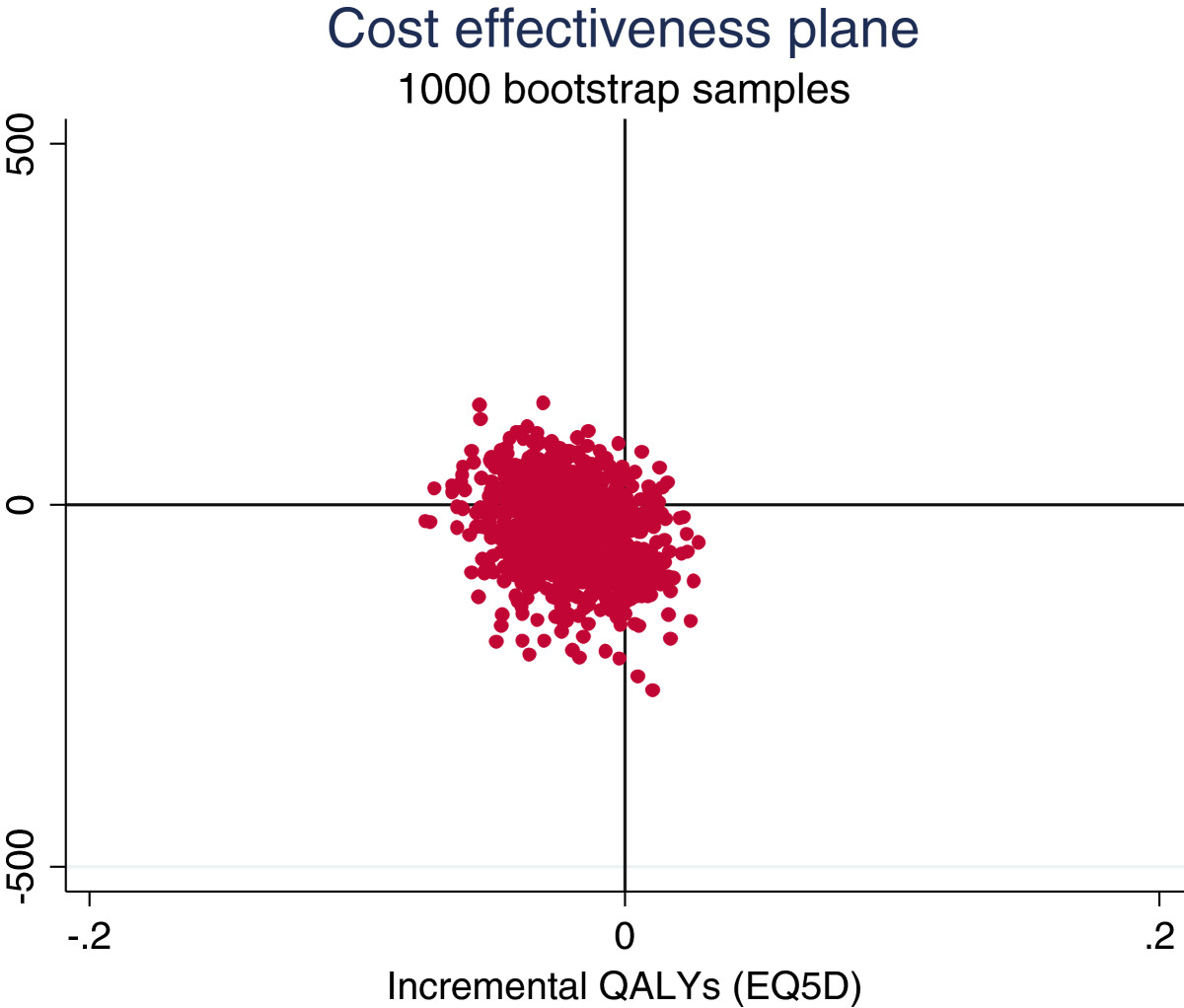
**Cost effectiveness plane.**

The regression-based estimates using imputed data and adjusting for baseline differences between the groups gave similar results: over four months, mean estimated costs were £38 cheaper but mean estimated QALYs were also 0.02 lower in the decision support arm than with usual care. These estimates give an incremental cost effectiveness ratio of £1900 (£38/0.02) per QALY gained for usual care compared with the decision support package, suggesting that the decision support intervention would not be cost-effective. The extent of uncertainty over the cost-effectiveness results is illustrated by the cost-effectiveness plane and the cost effectiveness acceptability curve (Figure 3 and Additional file 4: Figure S1). They show that the decision support package is unlikely to be cost-effective: with only 16% probability of being cost-effective at a threshold of £20,000 per QALY gained. The expected value of perfect information estimated from our base case is £30 per patient and £67 per patient when costs and QALYs are extrapolated up to one year from baseline.

## Discussion

Despite having a carefully designed decision support package we have failed to show an improvement in satisfaction with treatment. The striking finding here is that for our primary outcome and for all but one of our secondary outcomes the direction of change is neutral or favours the control arm. For one of these; the Roland Morris Disability Questionnaire this difference appears clinically important. The Roland Morris Disability Questionnaire is the leading primary outcome in community based back pain studies [47]. The point estimate of the dis-benefit from the decision support package at 2.3 points (95% CI 0.1 to 4.5) is larger than the effect size found in the definitive trials supporting the use of the back pain treatments included in the decision support package. Previous back pain trials have reported a net benefit on the Roland Morris Disability Questionnaire at three months of 1.4 (95% CI 0.6 to 2.1) for an exercise programme, 1.6 (95% CI 0.8 to 2.3) for a manipulation package and 1.1 points, (0.37 to 1.74) for a group cognitive behavioural approach [48, 49]. This means that the beneficial effects of physiotherapy treatment might be negated by our decision support package.

One of the main strengths of this trial is the rigorous design of the intervention package; that has itself been subject to external peer review [32]. The intervention was developed systematically using the International Patient Decision Aid Standards Collaboration (IPDAS) framework and its related checklist [50, 51]. These cover best practice in the development of decision support packages. The training package for the physiotherapist was grounded in principles from the Calgary-Cambridge Guidelines [52]. The training was designed and delivered by a Health Psychologist (HS) with expertise in communication skills.

Some caution is needed in interpreting these data because this is a pilot study run in one community physiotherapy service thus the results may not be generalisable. Nevertheless, the study was still adequately powered to test the effect of our intervention on treatment satisfaction. It is one of a very small number of trials of decision aids that have collected patient reported outcomes and the only trial, of which we are aware, to have reported a cost-effectiveness analysis [13]. Some caution is needed in interpreting the economic data because of the large amount of missing data. Nevertheless, we have demonstrated that our intervention does not improve satisfaction, it may produce worse clinical outcomes, and that it is unlikely to be a cost-effective option.

That we were not able to randomise those therapists joining the department after the initial training compromises our randomisation potentially leading to bias. That the allocation of patients to physiotherapists was managed by the department’s appointment staff blind to randomisation gives some reassurance that the two groups should have been well matched for unmeasured confounders at baseline. Since new appointees tended to be more inexperienced we judged that the direction of any bias would be towards reducing any positive effect size. In light of our results we did an additional post-hoc sensitivity analysis, for the RMDQ, excluding participants treated by physiotherapists recruited after randomisation. This showed the between group difference in the RMDQ of 3.11 (95% CI 0.51 – 5.71). This does not suggest that any bias introduced would substantially affect our conclusions (Additional file 5: Table S3).

The 2011 Cochrane review of decision aids did not identify any apparent adverse effects on health outcomes or satisfaction in 86 trials of decision support [11]. The authors did note the risk of bias from possible failure to report negative outcomes in published studies. Our data are not sufficient to conclude unequivocally that this decision support package is harmful. The harm identified might reflect no more than random chance in a study with multiple outcomes. Nevertheless, the overall trend across our pool of outcomes is to favour the control intervention and the harm identified was in a measure which would have been likely to be chosen as a primary outcome in any definitive trial. It would be difficult to justify a further randomised trial to confirm our findings.

We hypothesise that this decision support package introduced uncertainty about the overall effectiveness of the available treatment options, both in terms of their modest effect sizes and the weakness of the underpinning evidence, thus reducing expectation of benefit. Our promotion of evidence based practice and shared decision making may have affected the therapist-patient interaction, physiotherapist’s confidence in the treatment, or the patients’ confidence in the physiotherapist thus reducing the therapeutic effectiveness of the encounter. There is, for example, evidence that acupuncture is more effective when delivered by an enthusiastic practitioner rather than a neutral or negative practitioner [53]. In this trial we did not include a process evaluation that might have shed light on these issues. Future trials of similar tools should include such an evaluation.

## Conclusion

Although our findings are specific to our approach to improving choice of back pain treatments, our findings may be of considerable importance more generally. The way in which risk information is presented can influence decisions made [54]. Our results add to the evidence that it cannot be assumed that the provision of additional information and support for patients to achieve informed decisions is risk free. Before decision aids are implemented they should be formally evaluated to ensure safety as well as efficacy and cost-effectiveness.

## Electronic supplementary material

Additional file 1:Intervention Booklet.(PDF 540 KB)

Additional file 2: Table S1: Unit costs for NHS services, tests and drugs. (DOCX 17 KB)

Additional file 3: Table S2: Mean NHS costs (£): 0 – 4 months. (DOCX 18 KB)

Additional file 4: Figure S1: Cost effectiveness acceptability curve. (DOCX 22 KB)

Additional file 5: Table S3: Analysis of primary and secondary outcomes excluding participants seen by physiotherapists who joined post randomisation. Proportions and relative risk (usual care against decision support package) for the primary outcome, satisfaction with treatments. Mean change from baseline and difference between treatments for secondary outcomes. All estimates with 95% confidence interval. (DOCX 16 KB)

Below are the links to the authors’ original submitted files for images.

Authors’ original file for figure 1

Authors’ original file for figure 2

Authors’ original file for figure 3

## Abbreviations

QALs: Quality adjusted life years
NHS: National Health Service
NICE: National Institute for Health and Care Excellence
RCT: Randomised controlled trial
SF-12: Short form 12
EQ-5D: EuroQol-5 dimensions
RMDQ: Roland Morris disability questionnaire
ICC: Intra-cluster correlation coefficient
MICE: Multivariate imputation using chained equations
IPDAS: International patient decision aid standards collaboration.
