# Supplementary material for: Primum non nocere: shared informed decision making in low back pain – a pilot cluster randomised trial
Source: BMC Musculoskelet Disord. 2014 Aug 21;15:282. doi: 10.1186/1471-2474-15-282 (PMC4247192; doi:10.1186/1471-2474-15-282)
Supplement: Supplementary file 1 — Additional file 1:Intervention Booklet.(PDF 540 KB) [file 12891_2014_2315_MOESM1_ESM.pdf]

IMPACT LBP

IMPROVING PATIENT CHOICE IN TREATING LOW BACK PAIN

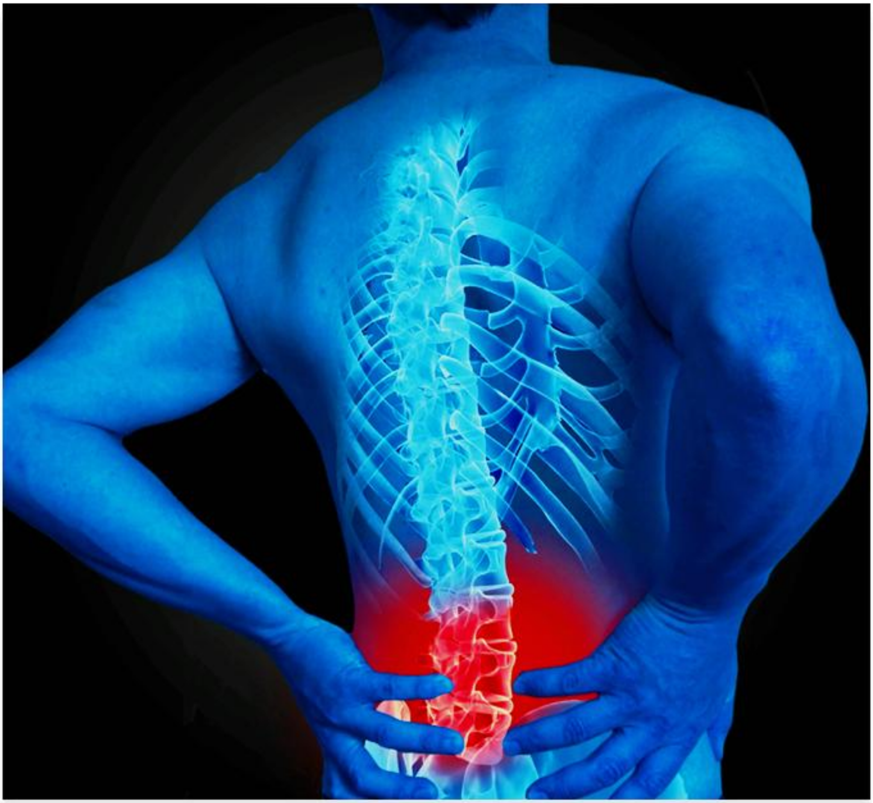

Choosing your Back Pain Treatment

Warwick Medical School
CLINICAL TRIALS UNIT

About this booklet

This booklet has been developed by a team of leading researchers and expert clinicians in decision making and low back pain. It has been developed as part of a research project, funded by the National Institute of Health Research-Research for Patient Benefit Programme. The project aims to help improve patient satisfaction with their choice of back pain treatment.

None of the authors or their associates stands to gain or lose by choices patients make after using this decision aid.

Views expressed here are those of the authors and do not necessarily reflect those of the National Institute for Health Research.

Further information:

- For more information on the funder visit: http://www.nihr.ac.uk/Pages/default.aspx
- For more information on the project visit: http://www2.warwick.ac.uk/fac/med/research/ctu/trials/ecr/impact-lbp/

Decision Support Package Version 1.0/06 May 2011
© Copyright Warwick Medical School, Clinical Trials Unit, Gibbet Hill Road, CV4 7AL

With thanks to the Wolfson Foundation and Advantage West Midlands through Birmingham Science City Translational Medicine, Clinical Research and Infrastructure Trials Platform

This booklet may not be reproduced, republished, broadcasted or transmitted in any way without first obtaining permission from the copyright owner.

Welcome!

This booklet had been designed to help you decide and choose among the four treatments options for your low back pain. It contains information on the different treatment options available based on the findings and recommendations given by the 2009 National Institute of Health and Clinical Excellence (NICE) on the treatment and management of low back pain.

This booklet is for you if:

- you have persistent or recurrent low back pain that has lasted more than six weeks
- your GP or other health professional has said that your low back pain is not due to rare conditions such as cancer, infection, a fracture or an inflammatory disorder
- you have a GP referral to your local physiotherapy department

The main purpose of this booklet is to provide you with detailed information on five available treatment options for your low back pain provided by your local physiotherapy department. It also gives information on the resources you need in order to make a decision regarding your treatment choices and preferences.

This booklet is split into four sections:

1. What treatment options do I have?
2. What matters to me when choosing treatment?
3. What else do I need to know to make a decision?
4. What next?

As you read through this booklet, you may wish to discuss the information with others such as family members, friends, carers and/or other health professionals.

Low Back Pain

What is low back pain?

Low back pain is soreness or stiffness in the back, between the bottom of the rib cage and the top of the legs. Most people's low back pain is described as 'non-specific', meaning that it is caused by problems with structures in the back, rather than by rare conditions such as cancer, infection, a fracture or inflammation.

It is often difficult to pin point exactly which part of the back is causing a person's pain. What we do understand is that most back pain comes from the muscles, ligaments or joints in the back that can start to stiffen and become 'unfit'. Common symptoms of low back pain include: muscle spasms – involuntary tightening and relaxing of your muscles; pain that travels from your lower back down your buttocks and into your legs; and/or a reduced range of motion in any actions that involve your back, for example, you may find it difficult or painful to bend over to pick things up.

What treatment options do I have?

Whatever treatment option you select, remain physically active, and carry on with normal activities as much as possible.

NICE recommends that patients should be offered a choice of treatment options, taking into account their preference: an exercise programme, a course of manual therapy or a course of acupuncture, as well as advice on low back pain self-management only given by the physiotherapist.

Subsequent research has found that a cognitive behavioural approach is also effective in the management of low back pain. We have therefore included this as a treatment option.

Brief description of treatment options

TREATMENT OPTION	DESCRIPTION*
Structured group exercise programme	A supervised exercise programme which includes light physical exercise, muscle strengthening and stretching.<br><br>The exercise programme is available as:<br>a) a supervised group programme with up to eight people.<br>b) one-to-one sessions with a personal trainer.
Manual therapy	Manual therapy is delivered by the use of the therapist's hands to manipulate, mobilise or massage the tissues of the body.
Acupuncture needling	This involves the insertion of fine needles into the back, and other acupuncture points. It will also include twisting or other stimulation of needles during therapy.
Cognitive behavioural approach	This therapy deals with our response to pain by understanding the interaction of our thoughts, feelings, behaviours and the physical consequences of our response.
Advice on self management	This involves being given advice and information on self-management of low back pain by the physiotherapist.

***Full details of each treatment are provided in the next section of this booklet**

What are the general rates of improvement?
Many people with back pain improve without needing any additional treatment. Around half of people find that the effect their pain has on their life reduces noticeably over three months **without** treatment.

An additional 10-20% of people with non-specific low back pain find that the effect pain has on their lives improves substantially over three months after receiving any of these treatments. In other words, for most people with low back pain the outlook is good, however the available treatments will give additional benefit.

This means that for every 10 people, five will have noticeably less pain over three months without any treatment, and of the five remaining, one or two will gain additional benefit from any of the treatments. However, most people do continue with some pain.

For some, this means a **reduction** in the way pain affects their lives and this may include:

- having more independence in doing things for yourself
- less pain doing simple physical activities such as sitting, standing up and walking up stairs
- a less painful back generally
- a better appetite
- an ability to walk longer distances
- better sleep
- an ability to dress more easily
- managing to do housework
- being less ill tempered

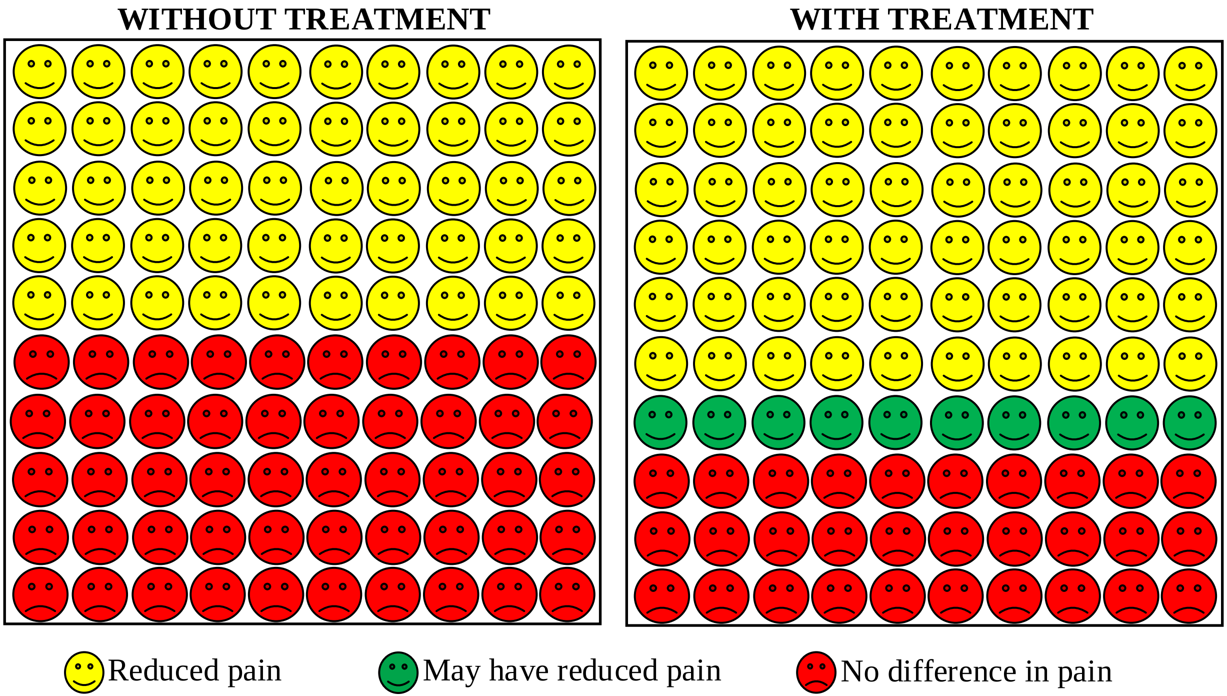

For every 100 people with back pain, 50 will get better without any treatment; 60 will get better with treatment and of the remaining 40, possibly 10 more may get better with treatment.

Where does this evidence come from?
The improvement levels discussed above have been obtained from high quality scientific research carried out with low back pain people, and the effect of any of these treatments on their pain.

What if I don't want any of these treatments?
Some patients may choose not to have treatment for their back pain. In most cases, back pain will usually get better on its own over time.

What will happen at my first appointment with the physiotherapist?
When you go for your first appointment with the physiotherapist, an assessment of your back will be carried out. You will then have the opportunity to discuss the treatment options available to you and your treatment preference with the physiotherapist. You will have the opportunity to ask questions. Following this, once a treatment decision is made, you will be booked in for that treatment.

Once you have been booked into any of the treatment sessions, you will have an ongoing review of your progress with your physiotherapist to discuss any issues/concerns you may have.

Depending on the results of the assessment, and the professional opinion of the physiotherapist, some/all of the above treatment options may be unsuitable for you. This will be discussed with you during your appointment.

Practitioner qualification
All physiotherapists in the department are qualified physiotherapists, registered with the Health Professions Council. A proportion of the staff members have post graduate Masters qualifications in delivery of the treatments. You will be treated by an appropriately trained therapist.

Location details
The physiotherapy department is based at Coventry and Warwickshire Hospital. All of the above mentioned treatment options will be available and delivered here.

Step 1: What treatment options do I have?

a) Structured Exercise Programme

What does a Structured Exercise Programme involve?
These structured exercise programmes may include a range of activities including: light physical exercises, muscle strengthening, postural control and stretching. All these therapeutic exercises are designed specifically for low back pain patients.

How does it work?
By increasing the muscle strength of your back and core muscles, and improving the range of movement of your joints, the back will be stronger and more mobile and thereby better able to function.

Will the exercise be personalised or general?
One-to-one sessions are personalised, group sessions are generalised with everyone doing similar therapeutic exercises. However, in group sessions, there is scope to tailor some of the exercise to enable you to work to your own ability.

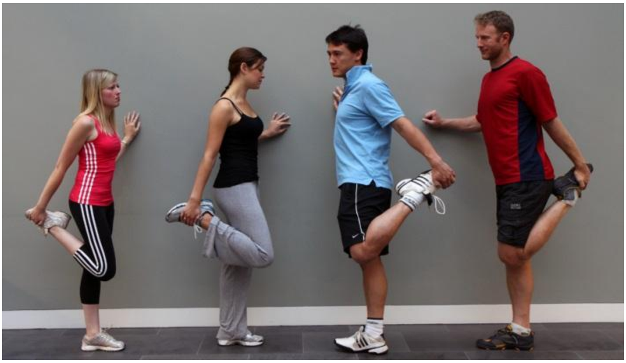

Group sessions will comprise of both men and women.

Will the sessions be supervised?

Yes, the group sessions and one-to-one sessions will be supervised by a physiotherapist and in some cases a personal trainer as well.

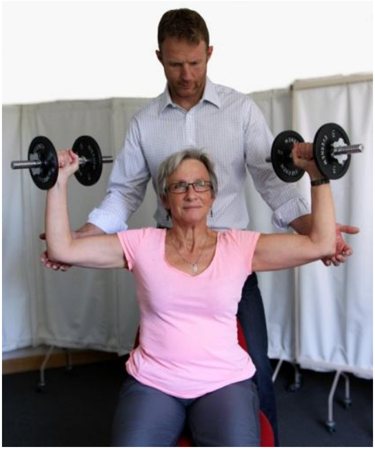

What is the number of staff to patient ratio?

In a group session, there will be a maximum number of eight people with two members of staff - a physiotherapist and personal trainer.

In a one-to-one session, you will work with a personal trainer.

What will be the level of difficulty?

The personal trainers and physiotherapists are there to encourage you to exercise, however you should work to your own abilities.

What should I wear?

As you will be exercising in the gym, please wear comfortable, appropriate sportswear and trainers.

Will I need to do the exercises at home?

Yes. As the main aim is to promote exercise to ease back pain, you are encouraged to practise exercises recommended to you by the physiotherapist or personal trainer at home about three or four times per week.

Will there be worksheets to monitor my progress?

Yes, every time you attend a gym session the therapist will provide you with a sheet to record what exercises you have done during the session, to monitor your progress.

How long is a course of structured exercise?
The group exercise and one to one sessions will last a maximum of six sessions, once a week.

You will be given a choice of either a structured group exercise programme, or one-to-one exercise sessions. You may select either of these depending on your preference. Both will be with a suitably qualified staff such as physiotherapists and personal trainers.

Will I need to attend all the sessions?
In order to gain maximum benefit from the group exercises, it is recommended that you attend all the sessions. There will be regular reviews with your physiotherapist and/or personal trainer on your progress.

What are the possible side effects of group exercise/one-to-one exercise?
Some patients have reported short term increase in their pain and/or discomfort. In most cases, this is because patients are doing muscle movements which they have not done for a long time, and the pain is generally not harmful to you.

How long is the waiting list?
At the end of your first assessment with the physiotherapist, you will be booked into the next available appointment structured group exercise programme, or one-to-one sessions depending on your preference.

b) Manual Therapy

The manual therapies include:

1. Spinal manipulation - applying pressure to joints in the spine then moving these joints beyond their normal range
2. Spinal mobilisation- moving joints within the normal range of movement
3. Massage of back muscles

Collectively these are all manual therapies - that is the therapist's hands deliver some, or all of the treatment.

What does the manual therapy involve?

Physiotherapists who are trained in manual therapy use a wide range of techniques, including short, sharp thrusts applied to the spine, gradually moving joints through a range of different positions, pulling or stretching muscles in a certain direction and/or application of a specifically directed manual thrust to a joint.

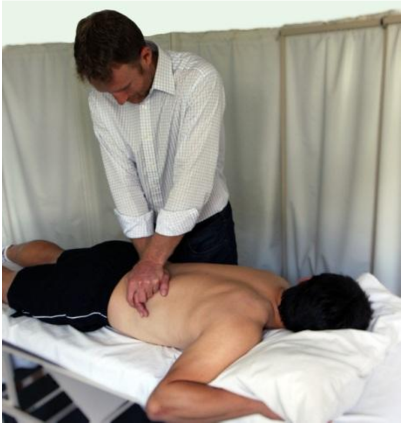

How does manual therapy work?

By increasing the range of joint movement and stretching muscles, manual therapy helps to treat painful soft tissue, providing pain relief.

What should I wear?

Please wear comfortable clothing, bearing in mind you will have to remove upper body clothing while keeping on any underwear during your treatment session.

Do I have to take my clothes off?
During the session, you may be asked to sit or lie down.

You will usually be asked to remove upper body clothing so the physiotherapist can access your spine.

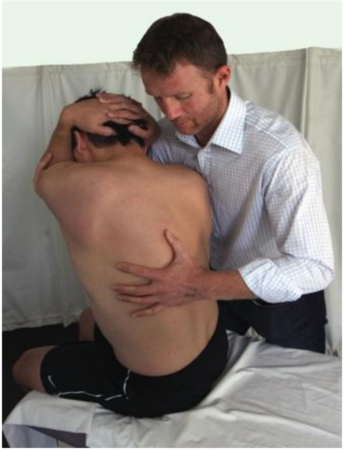

Will I need to do any exercises at home?
Yes, it may be part of the overall package. Your physiotherapist may recommend simple back muscle stretches, tailored to your needs that you may do at home to supplement the treatment you have received.

How long is a course of manual therapy?
The manual therapy course will comprise of up to a maximum of nine sessions over a period of up to 12 weeks. Each session typically lasts for up to 30 minutes.

Will I need to attend all sessions?
Yes, you are advised to attend all sessions (unless you recover after the first few sessions). However, if the treatment is not helping after a few sessions then the physiotherapist will discuss other low back pain treatment options.

Will it cause me any discomfort or pain?
Usually, manual therapy is not painful. If the therapist is treating an injury that is painful or inflamed, there may be some minor pain or discomfort. If you experience any pain or significant discomfort while having treatment, tell your therapist immediately.

What are the possible other side effects of manual therapy?
There is an extremely low risk of serious unwanted side effects when receiving spinal manipulation for low back pain. About half of patients receiving manual therapy will experience minor to moderate side effects such as temporary aches and pains, bruising and muscle soreness.

Some report soreness during or shortly after the treatment or experience an allergic reaction (e.g. rash or pimples) to the massage oil. Please inform your physiotherapist if you have ever experienced any allergic reactions to massage oils previously.

How long is the waiting list?
Typically, you will be booked in for your first manual therapy session seven days from your first consultation with the physiotherapist.

c) Acupuncture

Acupuncture refers to the insertion of fine needles into specific points in the body to produce a therapeutic effect. Acupuncture commonly includes manual stimulation of the needles, which is done by a fully trained therapist.

What does acupuncture involve?
Typically, an initial acupuncture session will involve taking your medical history, a physical examination and completion of a consent form before insertion of the acupuncture needles.

How does acupuncture work?
Acupuncture is thought to work by stimulating the brain and spinal cord to produce natural pain relieving chemicals. This ‘blocks’ some of the pain messages that your brain is receiving from your back.

Sometimes needles are inserted in the painful areas to directly reduce muscle spasm and/or relieve tenderness.

These effects, in turn, can lead to increased movement and function of the painful area. The needles are inserted into specific places on the body, which practitioners call 'acupuncture points'.

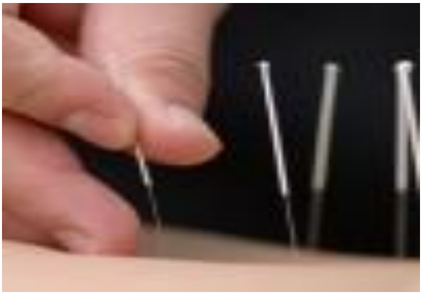

When is acupuncture not appropriate?
Acupuncture is not suitable for you if you have:

- an unstable heart condition
- poorly controlled epilepsy or diabetes
- a known stainless steel or nickel allergy
- a severe phobia of needles
- an infection in the area to be needled

If you fall into any of these categories please let your physiotherapist know, as it is unlikely that acupuncture will be appropriate for you.

Are the needles sterile?
Yes, acupuncture involves using single use, pre-sterilised, disposable needles of varying widths, lengths and materials that pierce the skin at the acupuncture points.

How long are the needles in for?
The needles are typically left in position for some 20-30 minutes before being removed.

How long are the needles?
The needles used are fine and can vary in length from 3cm to 8cm. The length of needles used will depend on where they are being inserted.

How many needles will be used in a session?
This will vary according to each patient and the nature of back pain. Typically between two and 10 needles will be used.

Will I sit or stand?
During the session, you will usually be asked to sit or lie down. You may also be asked to remove some clothes, so that the therapist can access the relevant places on your body.

Where do the needles go?
The needles may be places in and around the painful area, away from the painful area (e.g. in your hands and feet) or on the opposite side of the body. This is because needling away from the painful area can be effective. The physiotherapist will determine which ‘acupuncture points’ to use.

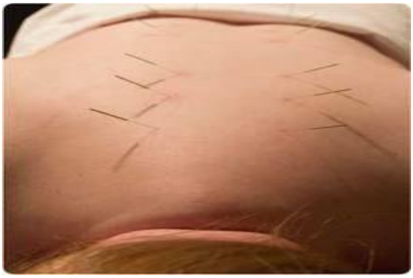

What should I wear?
Please wear comfortable clothing, bearing in mind you will have to remove upper body clothing while keeping on any underwear during your treatment session.

Can I drive afterwards?
Yes, you can drive after your acupuncture session. However, if you experience any post drowsiness after your treatment session, you are advised not to drive immediately.

How long is a course of acupuncture?
A course of acupuncture will comprise of up to a maximum of ten sessions over a period of up to 12 weeks. You will be booked in for a course of acupuncture at the acupuncture clinic that runs weekly at the physiotherapy department.

You may be offered one or two sessions in a week, and the frequency reduces over time to fortnightly or monthly.

What happens at the first acupuncture session?
The first session will be a ‘taster’ for 10-15minutes to assess your suitability for the acupuncture.

Make sure you have had something to eat within two hours of your appointment time, as there is increased risk of fainting or drowsiness if you have not eaten anything. The second session, if there were no unfavourable reactions, will be for about 30 minutes.

Will I need to attend all sessions?
If you agree with the physiotherapist that acupuncture is suitable for you after the first ‘taster session’, you will be advised to attend the full course of acupuncture treatment (unless you recover after the first few sessions). However, if the treatment is not helping after a few sessions then the physiotherapist will discuss other low back pain treatment options.

Will it cause me any discomfort or pain?
There will be a brief sharp sensation felt as the needle penetrates the skin. Some patients have reported tingling and numbness, as well as local soreness at the acupuncture points; whilst others have reported no symptoms at all. These sensations are unique to each individual.

What are the side effects of acupuncture?
Side effects reported by some patients include post-treatment drowsiness, minor bleeding or bruising, discomfort at the needle site, and fainting in rare circumstances. Some have also experienced temporary increase in pain. This is usually a good sign that further acupuncture sessions may result in an improvement.

How long is the waiting list?
You will normally be booked in for the next available slot at the acupuncture clinic that runs weekly.

d) Cognitive Behavioural Approach

Cognitive behavioural approach (CBA) aims to help patients change the way they think, feel and behave. This is because all of these factors can affect how pain is experienced by individuals.

What does CBA involve?

Before you start your sessions, you will be invited to attend a seminar which introduces self-management principles. This will be a discussion on what CBA is, what it involves and what may be expected from you during the seminar. After this initial seminar, you will need to decide if you wish to proceed with the full CBA programme.

Each session will be composed of an informal, relaxed educational talk and discussion followed by exercises. You will not be forced to speak, however the therapist will encourage all members of the group to participate.

At the end of each session you will be given a summary of the information.

How does CBA work?

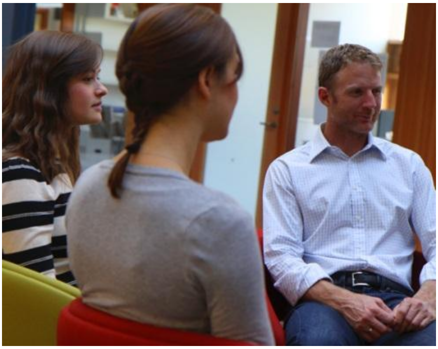

By dealing with thoughts, feelings and beliefs, CBA can help you to change how you think ('cognitive') and what you do ('behaviour'), which can help you feel better about life.

It can help people who have long-term conditions to cope better with their back pain symptoms, and manage the pain more effectively.

Will the information I provide in the group be kept confidential?
Yes, as part of the group ground rules we are keen for all patients to be aware of confidentiality. Confidential issues discussed in the session should not be divulged outside of the session.

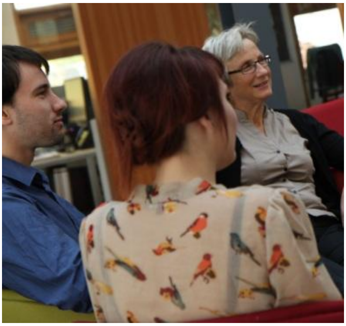

How many participants will there be in the group?
The course will consist of a maximum of 8 people, both men and women.

Can I stand during sessions to alleviate my pain?
Yes, you are free to stand and stretch as required during the session. You may also bring any items which will make sitting easier for you e.g. a cushion.

Will there be worksheets to monitor my progress?
Yes there will be homework sheets to help monitor your progress.

How long is a course of CBA?
CBA is offered as a group therapy, with others who have back pain. You will be offered a total of six sessions, one session per week lasting between 1½ to 2 hours. There will be a week's break between sessions three and four.

Will I need to attend all sessions?
Yes, you will be expected to attend all the weekly sessions of CBA until the end of the course. If, however, you have any concerns over the suitability of CBA for you, please discuss this with your physiotherapist.

Are there any risks/side effects involved?
There are no known risks or side effects associated with this therapy, however certain discussions or topics may be upsetting for some patients.

How long is the waiting list?
The availability of this depends on the number of people who wish to join. The physiotherapist will get in touch with you to confirm when the next programme will be once the maximum number of 8 people for a group is reached.

e) Advice on self management

Depending on the results of your initial assessment with the consulting physiotherapist it may be most suitable to offer you advice on self-management only before discharge. The physiotherapist will discuss this with you during the consultation, give you advice and information to promote self-management of your low back pain, including information on the nature of non-specific low back pain.

What advice will I be given?
The physiotherapist will encourage you to be physically active and continue with normal activities as far as possible, recommending to you simple exercises that you may do at home that will help you cope with your low back pain.

Step 2: What matters to you when choosing treatment?

The table in the this section gives you the chance to write down what matters to you the most when choosing between these four treatments - the positive and negative reasons for any of the options.

Once you complete the columns this may help you decide which treatment you wish to have.

You may wish to discuss this with others and the physiotherapist at your first appointment.

Any other reasons:

____

____

____

____

____

____

____

____

____

____

____

____

**TREATMENT OPTION**	**POSITIVE** (reasons to choose treatment)	**NEGATIVE** (reasons to avoid treatment)
**Structured exercise programme**		
**Manual therapy**		
**Acupuncture**		
**Cognitive behavioural approach**		
**Advice on self-management and discharge**		

Step 3: What else do I need to know to make a decision?

Knowledge:

Are you clear about the treatment options available to you?

☐ **Yes** ☐ **No**

Values:

From the table you have completed with the positive and negative aspects of the treatment options, are you clear about which factors matter the most to you?

☐ **Yes** ☐ **No**

Support:

Do you have enough support and advice to make a treatment choice?

☐ **Yes** ☐ **No**

Choice:

Do you feel confident about the treatment option that may be suitable for you?

☐ **Yes** ☐ **No**

You are encouraged to discuss the above needs for decision making with friends, family and/or carers before your consultation, and with your physiotherapist during your consultation. If you have any queries or concerns, please list them in the next section.

Step 4: What next?

How sure are you about making a treatment decision for your low back pain?

☐ I have made my decision about which treatment option I would prefer and would like to discuss this with the physiotherapist. State here preferred treatment:

____

☐ I am not sure, I would like to discuss my options with the physiotherapist.

Please list any questions or concerns that you may have or you would like to discuss with your physiotherapist during your consultation:

____

____

____

____

____

____

____

____

____

Development of the Decision Support Package

Evidence Appraisal

Most of the information provided in this Decision Support Package was obtained from the 2009 National Institute of Health and Clinical Excellence (NICE) guidance on the early management of non-specific low back pain.

To develop the guidance provided in this booklet high quality evidence was reviewed, and treatments likely to be of benefit to patients were identified. Other sources of information have also been used.

The electronic sources of information used to develop this booklet can be accessed via http://www.warwick.ac.uk/go/impact-lbp. Alternatively hard copies are available on request.

Updating this booklet

This is the first version of this booklet. Future updates will be made when new research evidence is available from NICE.

Useful patient resources on chronic low back pain

2009 NICE Low Back Pain Guidelines: Information for people who use NHS services. http://www.nice.org.uk

Back Care www.backcare.org.uk - A national charity that aims to reduce the impact of back pain on society by providing information, support, promoting good practice and funding research. Helpline: 0845 130 2704

Pain Coalition www.paincoalition.org.uk -The Chronic Pain Policy Coalition is a newly established forum for patients, professionals and parliamentarians who operate at policy level to develop an improved strategy for the prevention, treatment and management of chronic pain and its associated conditions. Tel: 020 7202 8580

Action on Pain www.action-on-pain.co.uk – Provide advice and support to people living with pain and those affected by its intrusive effects on daily life. PainLine: 0845 6031593

The research team

Prof Martin Underwood MB ChB FRCGP MD
Professor of Primary Care Research, University of Warwick

Dr Shilpa Patel BSc MSc C.Psychol
Research Fellow, University of Warwick

Prof Jill Thistlethwaite MB BS FRCGP FRACGP PhD
Professor of Medical Education, University of Queensland

Prof Frances Griffiths MB ChB FRCGP PhD
Professor of Medicine in Society, University of Warwick

Dr Joanne Lord BSc MSc PhD
Reader in Health Economics, Health Economics Research Group (HERG), Brunel University

Mr Mark Woolvine BSc MSc
Extended Scope Practitioner Low Back Pain, Coventry Primary Care Trust, Coventry and Warwickshire Hospital

Prof Tim Friede Dipl.-Math PhD
Professor of Biostatistics, University Medical Center Göttingen

Ms Anne SW Ngunjiri BSc MPH
IMPACT-LBP Study Administrator, University of Warwick

Ms Sally Brown
Lay member, Universities/User Teaching and Research Action Partnership (UNTRAP)

Mr Colin Tysall
Lay member, Universities/User Teaching and Research Action Partnership (UNTRAP)
