# Supplementary material for: Primum non nocere: shared informed decision making in low back pain – a pilot cluster randomised trial
Source: BMC Musculoskelet Disord. 2014 Aug 21;15:282. doi: 10.1186/1471-2474-15-282 (PMC4247192; doi:10.1186/1471-2474-15-282)
Supplement: Supplementary file 2 — Additional file 2: Table S1: Unit costs for NHS services, tests and drugs. (DOCX 17 KB) [file 12891_2014_2315_MOESM2_ESM.docx]

Additional file 2: Table S1. Unit costs for NHS services, tests and drugs

|  | | | |
| --- | --- | --- | --- |
| **Item** | **Unit** | **Unit Cost (£)** | **Source Unit Cost** |
| NHS services: |  |  |  |
| General Practitioner | session | 45 | PSSRU 2011(p149) |
| Practice nurse | session | 13 | PSSRU 2011(p146) |
| Physiotherapist | session | 16 | PSSRU 2011 (p181) |
| Occupational Therapist | session | 56 | Ref cost: 651A |
| Doctor/nurse in an emergency  department(casualty) | session | 127 | PSSRU 2011(p91) |
| Hospital specialist (consultant or team  member) | contract hour | 162 | PSSRU 2011(p203) |
| Psychologist/counsellor | consultation | 60 | PSSRU 2011(p41) |
| Hospital stay | hospital day | 686 | PSSRU 2011(P91) |
| NHS tests: |  |  |  |
| X-rays | test | 5 | Assumed |
| CT scan | test | 109 | DH Ref cost 2011 - 2012(RA08A -RA14Z) |
| MRI scan | test | 157 | DH Ref cost 2011 - 2012(RA01A -RA07Z) |
| Blood tests | test | 3 | DH Ref cost (DAP823) |
| NHS drugs: |  |  |  |
| Pain killers | item | 4.2 | Weighted from NHS Prescription Costs Analysis |
| Anti-inflammatory drugs | item | 3.7 | Weighted from NHS Prescription Costs Analysis |
| Gels/creams | item | 5.4 | Weighted from NHS Prescription Costs Analysis |
| Sleeping pills | item | 1.6 | Weighted from NHS Prescription Costs Analysis |
| Anti-depressants | item | 1.3 | Weighted from NHS Prescription Costs Analysis |
| PSSRU: Unit Costs of Health and Social Care (PSSRU, [www.pssru.ac.uk](http://www.pssru.ac.uk)) | | | |
