# Supplementary material for: Primum non nocere: shared informed decision making in low back pain – a pilot cluster randomised trial
Source: BMC Musculoskelet Disord. 2014 Aug 21;15:282. doi: 10.1186/1471-2474-15-282 (PMC4247192; doi:10.1186/1471-2474-15-282)
Supplement: Supplementary file 3 — Additional file 3: Table S2: Mean NHS costs (£): 0 – 4 months. (DOCX 18 KB) [file 12891_2014_2315_MOESM3_ESM.docx]

Additional file 3: Table S2. Mean NHS costs (£): 0 – 4 months.

| **Type of care** | **Number of patients** | | **Mean cost, £ (SD)** | | **Mean treatment difference (95% CI)** |
| --- | --- | --- | --- | --- | --- |
|  | **DSP** | **Usual care** | **DSP** | **Usual care** | **(Usual care – DSP)** |
| NHS services: | 46 | 35 | 182.2  (275.5) | 206.9  (302.0) | 3.04  (-125.22 to 131.30) |
| General Practitioner | 57 | 39 | 63.2  (93.9) | 50.8  (67.1) | -12.39  (-47.08 to 22.30) |
| Practice nurse | 58 | 39 | 1.3  (5.3) | 0.3  (2.1) | -1.01  ( -2.78 to 0.75) |
| Physiotherapist visit* | 81 | 60 | 61.4  (6.5) | 49.9  (7.7) | -11.56  (-31.47 to 8.34) |
| Doctor/nurse in an emergency department (casualty) | 58 | 39 | 19.7  (88.4) | 6.5  (40.7) | -13.19  (-43.26 to 16.87) |
| Hospital specialist (consultant or team member) | 58 | 39 | 19.6  (80.7) | 87.2  (243.3) | 67.68  (-0.60 to 135.96) |
| Psychologist / counsellor | 58 | 39 | 5.6  (42.5) | 12.5  (77.8) | 6.88  (-17.48 to 31.23) |
| Hospital stay | 57 | 40 | 24.1  (127.3) | 0  (0) | -24.07  (-64.11 to 15.97) |
| NHS tests: | 57 | 39 | 22.1  (53.3) | 47.6  (106.4) | 25.54  (-7.14 to 58.22) |
| X-rays | 58 | 39 | 0.4  (1.4) | 0.3  (1.1) | -0.17  (-0.71 to 0 .36) |
| CT scan | 58 | 39 | 1.9  (14.3) | 2.8  (17.5) | 0.92  (-5.52 to 7.35) |
| MRI scan | 57 | 39 | 19.3  (52.0) | 44.3  (101.6) | 25  (-6.36 to 56.37) |
| Blood tests | 58 | 39 | 0.5  (1.4) | 0.3  (0.9) | -0.16  (-0.65 to 0.34) |
| NHS drugs: | 57 | 39 | 9.1  (11.7) | 9.4  (10.2) | 0.28  ( -4.30 to 4.87) |
| Pain killers | 57 | 39 | 4.9  (8.4) | 5.2  (6.3) | 0.34  (-2.81 to 3.48) |
| Anti-inflammatory drug | 58 | 39 | 2.9  (4.7) | 3.2  (4.3) | 0.29  (-1.58 to 2.16) |
| Gels/creams | 58 | 39 | 0.8  (2.8) | 0.3  (1.2) | -0.56  (-1.51 to 0.39) |
| Sleeping pills | 58 | 39 | 0.2  (0.9) | 0.2  (0.6) | -0.06  (-0.37 to 0.26) |
| Anti-depressants | 58 | 39 | 0.3  (1.0) | 0.5  (1.2) | 0.22  (-0.21 to 0.65) |
| Total NHS cost | 45 | 35 | 245.2  (314.4) | 271.0  (360.0) | 25.76  (-116.54 to 168.05) |
| Total cost ** | 45 | 35 | 264.7 (44.5) | 271.0  (360.0) | 6.32  (-136.00 to 148.61) |
| *Based on physiotherapist’s clinical record  ** Included intervention cost for the DSP arm  Abbreviations: DSP, decision support package; SD, standard deviation; CI, confidence interval. | | | | | |
