# Supplementary material for: Primum non nocere: shared informed decision making in low back pain – a pilot cluster randomised trial
Source: BMC Musculoskelet Disord. 2014 Aug 21;15:282. doi: 10.1186/1471-2474-15-282 (PMC4247192; doi:10.1186/1471-2474-15-282)
Supplement: Supplementary file 4 — Additional file 4: Figure S1: Cost effectiveness acceptability curve. (DOCX 22 KB) [file 12891_2014_2315_MOESM4_ESM.docx]

| **Additional file 4: Figure S1. Cost effectiveness acceptability curve**   \|  \| \| --- \| |
| --- | --- |
