# Supplementary material for: Primum non nocere: shared informed decision making in low back pain – a pilot cluster randomised trial
Source: BMC Musculoskelet Disord. 2014 Aug 21;15:282. doi: 10.1186/1471-2474-15-282 (PMC4247192; doi:10.1186/1471-2474-15-282)
Supplement: Supplementary file 5 — Additional file 5: Table S3: Analysis of primary and secondary outcomes excluding participants seen by physiotherapists who joined post randomisation. Proportions and relative risk (usual care against decision support package) for the primary outcome, satisfaction with treatments. Mean change from baseline and difference between treatments for secondary outcomes. All estimates with 95% confidence interval. (DOCX 16 KB) [file 12891_2014_2315_MOESM5_ESM.docx]

Additional file 5: Table S3. Analysis of primary and secondary outcomes excluding participants seen by physiotherapists who joined post randomisation. Proportions and relative risk (usual care against decision support package) for the primary outcome, satisfaction with treatments. Mean change from baseline and difference between treatments for secondary outcomes. All estimates with 95% confidence interval.

|  | Decision support package | | Usual care | |  | |
| --- | --- | --- | --- | --- | --- | --- |
| *Primary outcome* | | | | | | |
|  | Proportion | | | | Relative risk | |
| Satisfaction with treatment | | | | | | |
|  | 0.5 | (0.04 to 1.0) | 0.8 | (0.01 to 1.0) | 1.45 | (0.85 to 2.49) |
| *Secondary outcomes* | | | | | | |
|  | Mean change from baseline | | | | Mean treatment difference* | |
| Roland Morris disability questionnaire (positive change=improvement) | | | | | | |
|  | 2.0 | (0.5 to 3.4) | 5.1 | (2.9 to 7.3) | -3.11 | (-5.71 to -0.51) |
| Modified Von Korff disability score (positive change=improvement) | | | | | | |
|  | 17.1 | (11.0 to 23.3) | 24.3 | (15.3 to 33.3) | -7.20 | (-18.00 to 3.60) |
| Modified Von Korff pain score (positive change=improvement) | | | | | | |
|  | 14.9 | (7.0 to 22.8) | 26.4 | (16.1 to 36.7) | -11.55 | (-24.46 to 1.36) |
| Norm-based physical component score of SF-12 (negative change=improvement) | | | | | | |
|  | -5.6 | (-7.6 to -3.5) | -9.0 | (-12.2 to -5.9) | 3.45 | (-0.30 to 7.19) |
| Norm-based mental component score of SF-12 (negative change=improvement) | | | | | | |
|  | -3.5 | (-6.6 to -0.4) | -0.9 | (-5.6 to 3.8) | -2.59 | (-8.15 to 2.97) |
| Hospital anxiety and depression scale: anxiety (positive change=improvement) | | | | | | |
|  | 0.5 | (-0.5 to 1.6) | 1.3 | (-0.3 to 2.9) | -0.81 | (-2.68 to 1.07) |
| Hospital anxiety and depression scale: depression (positive change=improvement) | | | | | | |
|  | 1.1 | (0.01 to 2.2) | 2.8 | (1.3 to 4.3) | -1.67 | (-3.29 to -0.06) |
| Pain self-efficacy questionnaire (negative change=improvement) | | | | | | |
|  | -4.8 | (-10.3 to 0.8) | -9.6 | (-16.7 to -2.4) | 4.80 | (-3.47 to 13.07) |
| Fear avoidance beliefs questionnaire (positive change=improvement) | | | | | | |
|  | 2.0 | (-0.04 to 4.0) | 3.5 | (0.4 to 6.6) | -1.53 | (-5.09 to 2.03) |
| * Mean difference = (decision support package – usual care) after adjusting for years of experience and pain severity at baseline as fixed effects and physiotherapist as random effects; negative difference = favours usual care | | | | | | |
