# Supplementary figures and images for: Primum non nocere: shared informed decision making in low back pain – a pilot cluster randomised trial
Source: BMC Musculoskelet Disord. 2014 Aug 21;15:282. doi: 10.1186/1471-2474-15-282 (PMC4247192; doi:10.1186/1471-2474-15-282)

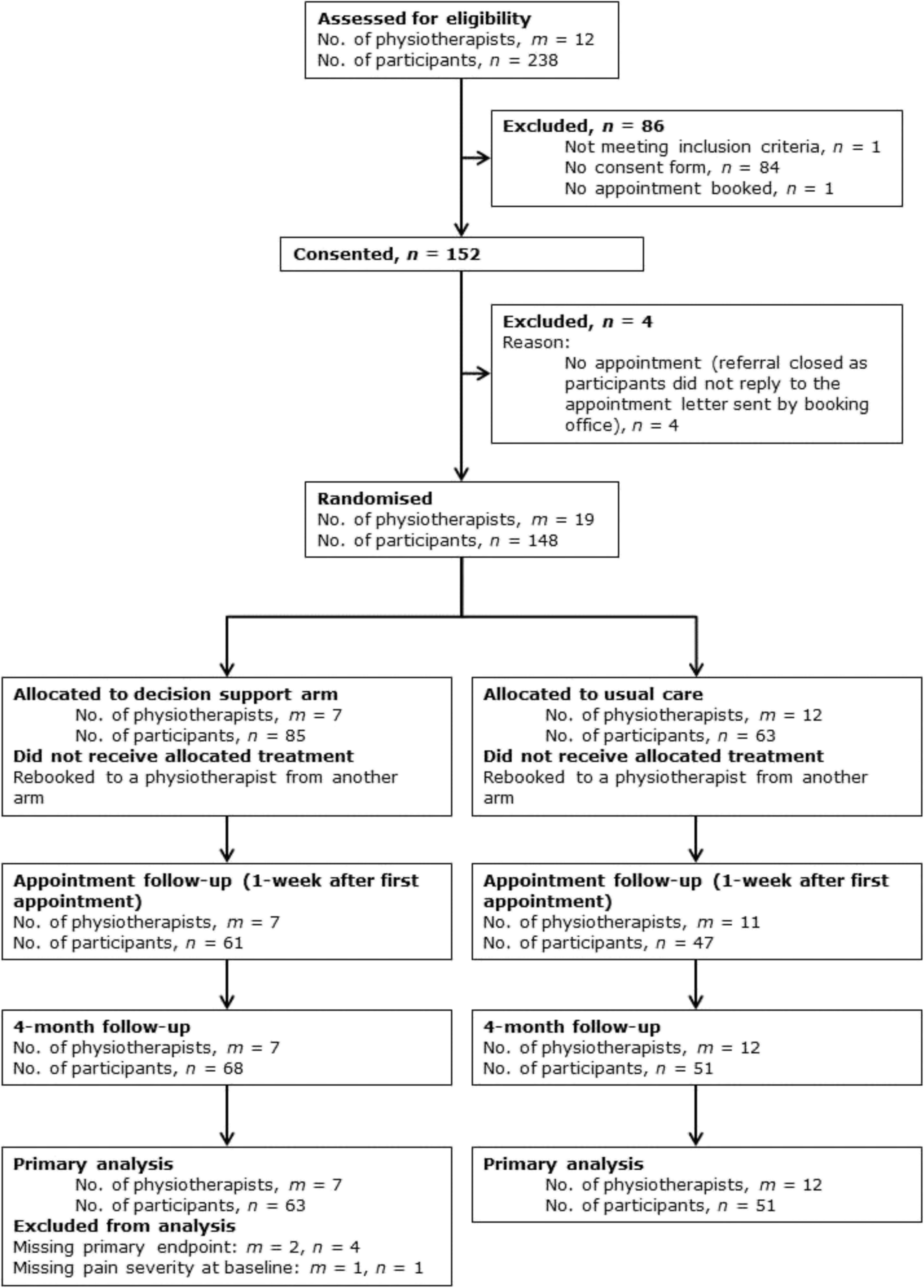

Supplement: Supplementary file 6 — Authors’ original file for figure 1 [file 12891_2014_2315_MOESM6_ESM.tif]

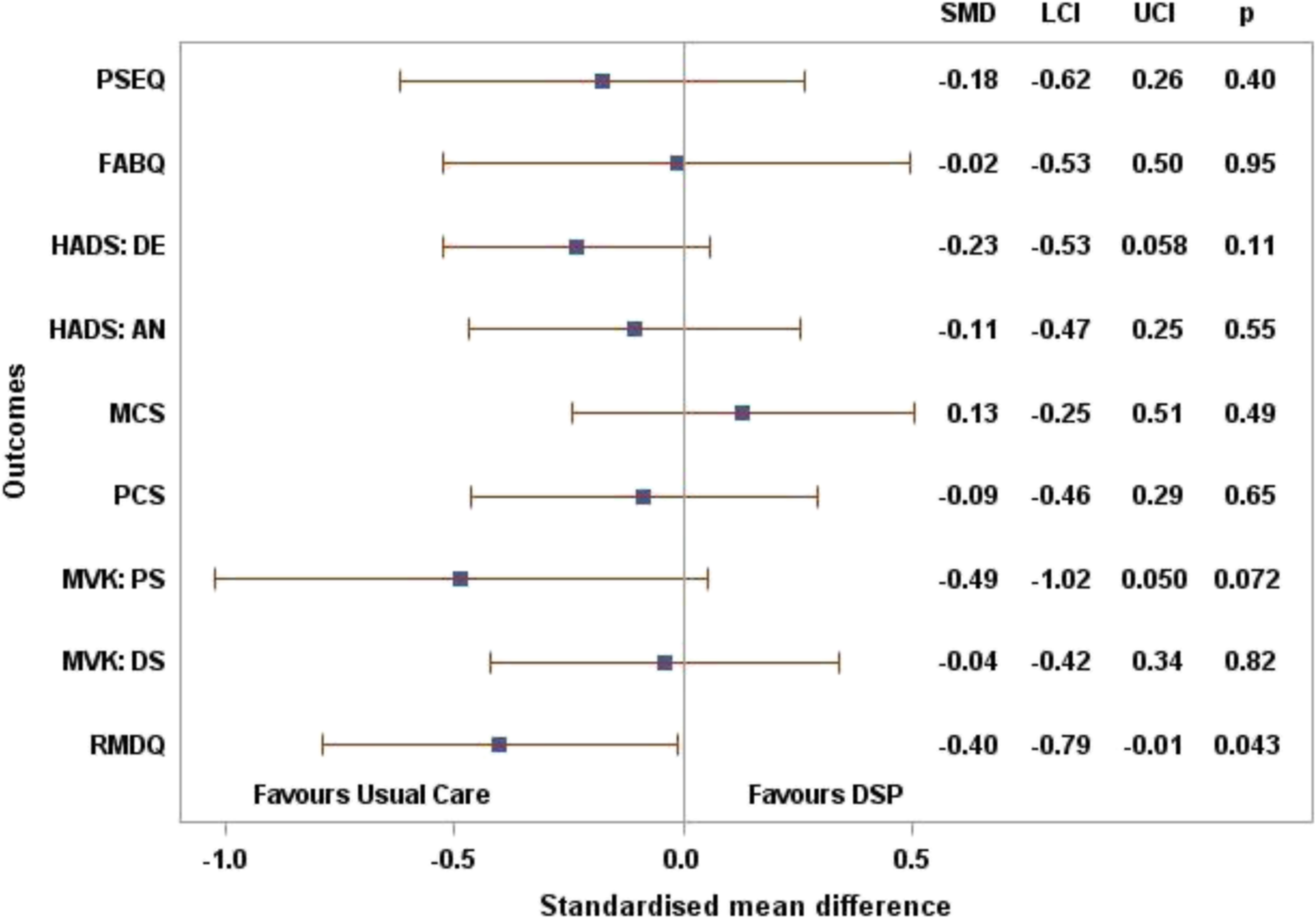

Supplement: Supplementary file 7 — Authors’ original file for figure 2 [file 12891_2014_2315_MOESM7_ESM.tif]

# Cost effectiveness plane

1000 bootstrap samples

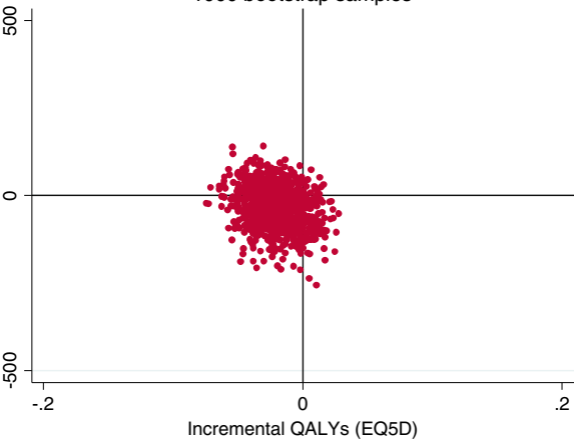

Supplement: Supplementary file 8 — Authors’ original file for figure 3 [file 12891_2014_2315_MOESM8_ESM.pdf]
